# Association of the Use of the Mobile Phone with Physical Fitness and Academic Performance: A Cross-Sectional Study

**DOI:** 10.3390/ijerph18031042

**Published:** 2021-01-25

**Authors:** Alfredo Bravo-Sánchez, Javier Morán-García, Pablo Abián, Javier Abián-Vicén

**Affiliations:** 1 Performance and Sport Rehabilitation Laboratory, Faculty of Sport Sciences, University of Castilla-La Mancha, 45071 Toledo, Spain; alfredo.bravo@uclm.es (A.B.-S.); javimg5@hotmail.com (J.M.-G.); 2 Faculty of Humanities and Social Sciences, Comillas Pontifical University, 28049 Madrid, Spain; pabloo9@hotmail.com

**Keywords:** physical activity, adolescents, cell phone use, educational test performance, gender-related differences

## Abstract

The aim of this study was to analyse the association of the use of the mobile phone with physical fitness (PF) and academic performance in secondary school students and its gender-related differences. A total of 501 high school students participated in the study (236 girls and 265 boys; 12–18 years). Use of the mobile phone and sample distributions were done with the Mobile-Related Experience Questionnaire (CERM): low use of mobile phone (LMP = 10–15 points), medium use of mobile phone (MMP = 16–23 points) and high use of mobile phone (HMP = 24–40 points). PF via Eurofit test battery and academic performance were recorded, and gender was used as a differentiating factor. The HMP group registered lower values than the LMP group for academic performance (Spanish: 4.78 ± 2.26 vs. 3.90 ± 1.96 points; *p* = 0.007, Mathematics: 4.91 ± 2.23 vs. 4.00 ± 1.84 points; *p* = 0.007) and PF (Abdominals: 6.83 ± 2.40 vs. 5.41 ± 2.46 points; *p* < 0.001, Broad jump: 6.24 ± 3.02 vs. 4.94 ± 2.28 points; *p* = 0.013). The boy students showed greater values than girl students for PF in the LMP (medicine-ball-throw: 6.34 ± 2.24 vs. 5.28 ± 1.86 points, *p* = 0.007) and MMP (medicine-ball-throw: 6.49 ± 2.52 vs. 5.02 ± 1.68 points; *p* < 0.001) groups, but no gender-related differences were found in the HMP group. In conclusion, high use of the mobile phone was related to worse results in the PF tests and academic performance. Gender-related differences were found for academic performance regardless of the use of the mobile, but for physical fitness no gender differences were found in HMP group.

## 1. Introduction

The advent of new technologies, above all the smartphone, has meant a considerable change in the lives of people in general and especially in adolescents. The latter deserve special attention in terms of their relationship with these technologies that are present in their daily lives [1]. Currently, more than 60% of adolescents have a mobile, and it is commonly used in their activity time [2]. Thus, they tend to be more vulnerable to suffering from problematic situations such as negative feelings or low self-esteem when they do not have a mobile phone or do not receive messages or calls [3]. In addition, the reduction in time spent on physical activity and studying in favour of mobile phone use could be related to poor development in the adolescent’s academic and physical area [4]. Therefore, adolescence is considered an important risk factor due to the high frequency of Internet connection and mobile phone use [5]. 

Physical fitness is defined as a set of attributes related to health [6] and the level of physical fitness is considered a powerful indicator of health in adolescence [7]. Physical fitness values in Spain are somewhat lower compared to other countries [8]. This may be due to the decrease in the practice of physical activity that occurs especially in adolescence as shown in previous research [9]. Physical inactivity or the decrease of physical activity during adolescence is related to lower values of physical fitness, to the detriment of health and quality of life, as well as the development of cardiovascular diseases, obesity and osteoporosis during adulthood [10]. In addition, the use of a smartphone could contribute to the decrease in physical activity and, therefore, the reduction of physical fitness levels [11]. Most of the research has been focused on the use of the Internet, apps and smartphones as a solution for combatting sedentarism [12], or other factors such as academic performance [13], although the relationship between the use of the mobile phone and the risk of sedentarism has been less reported [14]. High school may become the only real option for performing physical activity for adolescents [15], and therefore it is also a favourable environment for evaluating physical fitness and adopting possible measures that may prevent diseases in adulthood.

Academic performance could be defined as the students’ performance in each year, which is rated on a point scale. In relation to the physical fitness of high school students, the best values of academic performance during adolescence used to be associated with good health and low body fat in adulthood [16]. Furthermore, other studies point out that on the one hand adolescents who have physical activity habits or practice sports at a competitive level obtain better academic results than sedentary students [17,18], and on the other hand, spending more than two hours per day in front of a screen could be associated with lower academic achievement among school-aged children [19]. Some research showed differences between boy and girl students in physical fitness and academic performance; while boys recorded better physical fitness performance [20], academic performance was higher for girls [21]. In addition, the relationship between leisure screen time and the probability of getting high academic performance has been reported in boy students but not in girl students [22].

Interest in the possible addiction of high school students to mobile phones has grown in recent years. Some studies have noted that adolescents with high mobile-phone dependence showed a greater risk of suffering psychological and social problems [23]. The Mobile-Related Experience Questionnaire (CERM) is a questionnaire based on 10 criteria and was designed to examine the degree of “addiction” to the mobile phone [24] showing higher values in female than male university students [25]. The consequences of the increase in mobile phone use in recent years and its gender-related differences should be studied in high school students. Therefore, the objective of this study was to analyse the association of the use of the mobile phone on physical fitness and academic performance in secondary school students and gender-related differences.

## 2. Materials and Methods 

### 2.1. Participants

**Figure 1:**
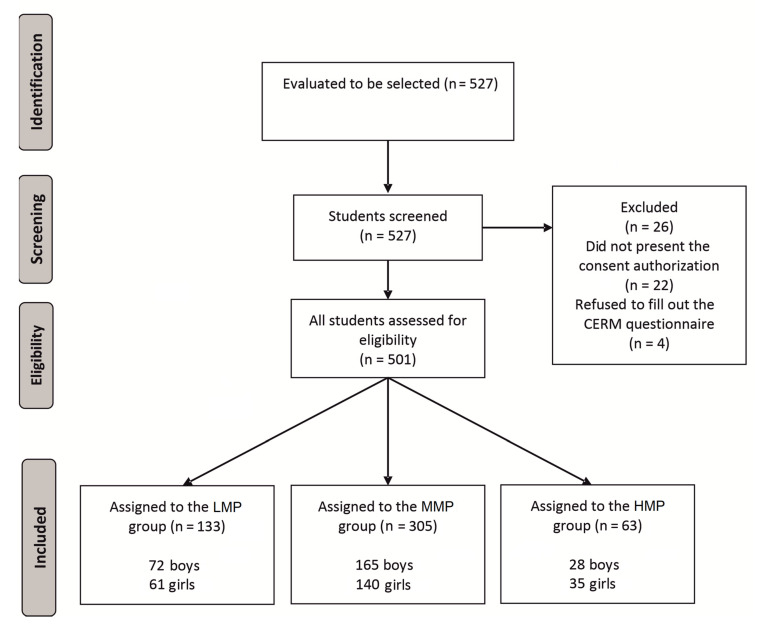
Flow chart of the study subjects. Mobile-Related Experience Questionnaire (CERM) = questionnaire about experiences related to the mobile phone; LMP = low use of mobile phone; MMP = medium use of the mobile phone; HMP = high use of the mobile phone.

A total of 501 secondary school students volunteered to participate in this cross-sectional and correlative study. Participants were divided into three groups according to their results in the CERM: low use of mobile phone (LMP), (N = 133, Age: 14.7 ± 1.4 years, Height: 163.9 ± 9.9 cm, Body Mass: 60.1 ± 14.3 kg, Body fat: 22.4 ± 10.8 %, mobile phone use: 13.5 ± 1.7 CERM points), medium use of mobile phone (MMP) (N = 305, Age: 15.2 ± 1.6 years, Height: 164.4 ± 11.9 cm, Body Mass: 59.9 ± 13.3 kg, Body fat: 21.7 ± 9.8%, mobile phone use: 18.7 ± 2.1 CERM points) and high use of mobile phone (HMP) (N = 63, Age: 15.3 ± 1.6 years, Height: 163.4 ± 11.8 cm, Body Mass: 59.4 ± 17.0 kg, Body fat: 24.4 ± 11.2%, mobile phone use: 25.7 ± 2.0 CERM points). The students came from state schools in the region of Castilla–La Mancha (Spain) (Figure 1).

All participants and their parents were informed in writing and verbally of the purpose and procedures of the investigation, and the parents of the participants provided a signed informed consent before the start of the study. The participants were free to leave the activity without the need to give any kind of explanation and without their departure implying any sanction. The study was approved by the Ethics Committee of Clinical Research at the Hospital Complex in Toledo (Spain) (number 487, dated 25 February 2020) according to the principles of the latest version of the Declaration of Helsinki.

### 2.2. Measure of Mobile Phone Use

The CERM was employed to divide the sample into 3 groups according to the points obtained [26]: LMP (total points were between 10 and 15 points, which means no problems derived from mobile phone use), MMP (total points were between 16 and 23 points, which means occasional problems derived from mobile phone use) and HMP (total points were between 24 and 40 points, which means problematic use of mobile phone). The questionnaire consists of 10 Likert items with four responses, classified from ‘1’ to ‘4’, in increasing order of intensity. It is used to analyse two mobile-related factors: problems due to emotional and communicational use, and conflicts related to mobile use. The CERM scale has good validity and reliability, and also showed a positive high correlation with the use of the mobile phone [24]. The CERM questionnaires were completed by each of the students at the beginning of the measurement process and the total score was used for further analysis. 

### 2.3. Physical Fitness Measurement

For the evaluation of physical fitness, we included several tests included in the Eurofit [27] which are validated and standardised by the Council of Europe and showed very good test-retest reliability and good criterion validity for adolescent evaluation [28]. The tests were applied by a researcher (JMG) in collaboration with the physical education teacher during a class after a familiarization session carried out on a different day. The marks obtained by the participants were evaluated according to their age and gender, and rated from 0 to 10 points, 10 being the highest qualification [29,30]. All tests were performed twice, and the best performance was chosen and expressed as a 0–10 mark. The test battery was applied in the following order:

- Abdominals in 1 min: it consisted of performing the highest number of trunk crunches in 1 min from a supine position on a mat and with the feet attached to a trellis. Repetitions to complete 1 min of exercise were recorded.
- 2 kg overhead medicine-ball throw: it is based on throwing a medicine ball over the head as far as possible by extension–flexion of the trunk and upper limbs. The medicine-ball throws were performed using a 2 kg rubber medicine ball. Each throw was measured for distance.
- Standing Long Jump Test (Broad jump). A tape measure was employed to measure the maximum horizontal distance jumped. The student stood behind a line marked on the ground with feet together. A 2-foot take-off and landing was performed, with a swing of the arms and bend of the knees to provide forward drive. The subject attempted to jump as far as possible, landing on both feet without falling backwards. Each jump was measured for distance.
- 50 m sprint: students performed 50 m runs at a maximum pace. Time to complete the 50 m run was recorded. The instructions given to start were a countdown from “ready, 3, 2, 1, go.”
- Trunk flexion test: it was based on performing a flexion of the body by bringing the arms back between the legs without bouncing. The objective was to analyse the flexibility of the trunk and lower extremities. Each attempt was measured for distance.
- 10 × 5 m shuttle run test (Agility test): Subject was required to run back and forth as fast as possible 10 times, along a 5 m course. Time to complete the agility test was recorded.

### 2.4. Academic Performance

To assess student achievement, we used their grade point average from the first trimester, provided by the participants and verified by the academic secretary of the corresponding centre. These grades summarise the student’s performance in the following common subjects: Spanish language and literature (Spanish), mathematics, English and physical education; they range from 0 (=very poor) to 10 (=excellent) and are standardised by Spanish law.

### 2.5. Statistical Analysis

The statistical analysis was performed with IBM SPSS Statistics 23.0 (SPSS, Chicago, IL, USA). All data are expressed as mean ± standard deviation. The data were tested for normality with a Kolmogorov–Smirnov test. Since the assumption of normality (all variables *p* > 0.05) was verified, a two-way ANOVA (2 × 3) was used to establish the differences in the CERM score, physical fitness and academic performance variables between the two gender groups (boys and girls) and among the three mobile-phone-use groups (LMP, MMP and HMP) and subsequently post-hoc Bonferroni’s tests were used when a significant main effect or an interaction between factors was found. The relationship between physical fitness tests and academic performance variables was analysed with a simple linear regression, from which the Pearson correlation coefficient was calculated. The effect size (ES) was calculated for all pairwise comparisons according to the formula proposed by Cohen [31]. The magnitude of the ES was interpreted using the scale of Cohen [31]: small (<0.2), medium (0.5) and large (>0.8). A probability level of *p* < 0.05 was defined as statistically significant.

## 3. Results

### 3.1. CERM Results

After the CERM questionnaire had been answered, the results of our study showed that 12.8% of the high school students had addictive behaviour in relation to mobile phone use. The number and the percentage of all groups of boy students assigned to risk groups (MMP and HMP) was higher than for girl students (68.8% boys and 64.2% girls). We did not find differences between boys’ and girls’ average results in the CERM questionnaire (boy vs. girl): 17.89 ± 4.01 vs. 18.61 ± 4.25 points; *p* = 0.055. No differences were described between boys’ and girls’ results in each group: LMP (13.32 ± 1.68 vs. 13.72 ± 1.64 points; *p* = 0.167); MMP (18.56 ± 2.02 vs. 18.95 ± 2.18 points; *p* = 0.110) and HMP (17.89 ± 4.01 vs. 18.61 ± 4.25 points; *p* = 0.055).

### 3.2. Physical Fitness

**Figure 2:**
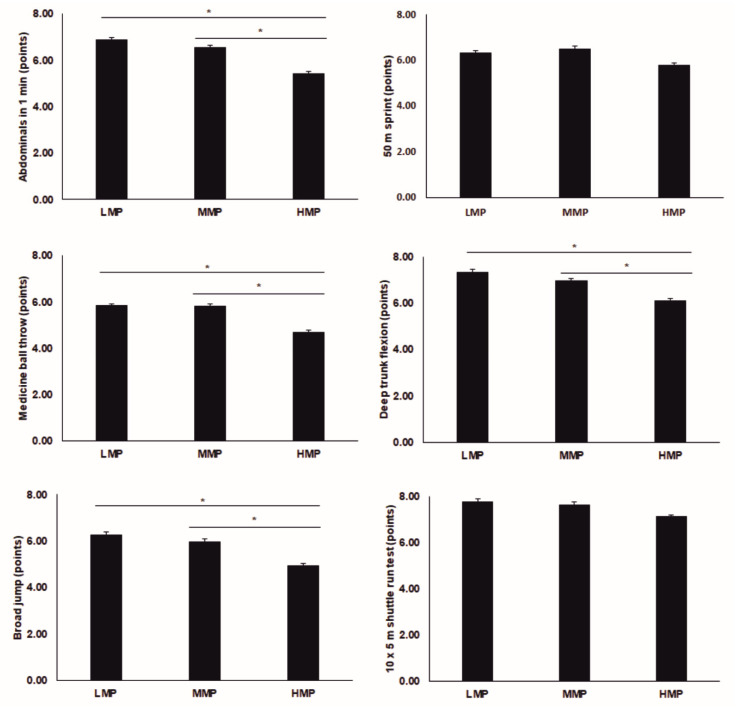
Results of physical fitness test. LMP = low use of the mobile phone; MMP = medium use of the mobile phone; HMP = high use of the mobile phone. * significant differences between groups; significant criteria *p* < 0.05.

The physical variables results are presented in Table 1. Significant interactions between use of mobile phone groups and gender were found for the abdominals test, medicine-ball throw, broad jump, 50 m sprint, trunk flexion test and agility test. The boys’ group showed higher values than the girls’ group in the LMP and MMP groups for the medicine ball (*p* < 0.008), broad jump (*p* < 0.003), 50 m sprint (*p* < 0.014) and agility (*p* < 0.025) tests. Boys also achieved higher scores than girls on abdominal tests for the MMP group (*p* < 0.001). No differences were found between boys and girls in the deep trunk-flexion test (*p* > 0.100). Finally, we did not find differences between the genders in HMP for any test (*p* > 0.129). Without the assumption of the gender effect, significant differences were described for the abdominals test (*p* = 0.001), medicine-ball throw (*p* = 0.001), broad jump (*p* = 0.011), and trunk-flexion test (*p* = 0.005) with higher values in the LMP and MMP than the HMP group (Figure 2).

Within the boys’ group, intermobile phone-use differences were observed in the PF performance being higher in the values of LMP group than HMP group for the abdominals test (*p* = 0.005), medicine-ball throw (*p* = 0.047), broad jump (*p* = 0.009) and trunk-flexion test (*p* = 0.047). In addition, higher values were also described for MMP than HMP in the abdominal test (*p* = 0.007), medicine-ball throw (*p* = 0.009) and trunk-flexion test (*p* = 0.004). No intergroup differences were found in the 50 m sprint and agility test (*p* > 0.05). For the girls’ group, no differences among mobile-use groups were found (*p* > 0.05).

### 3.3. Academic Performance

**Figure 3:**
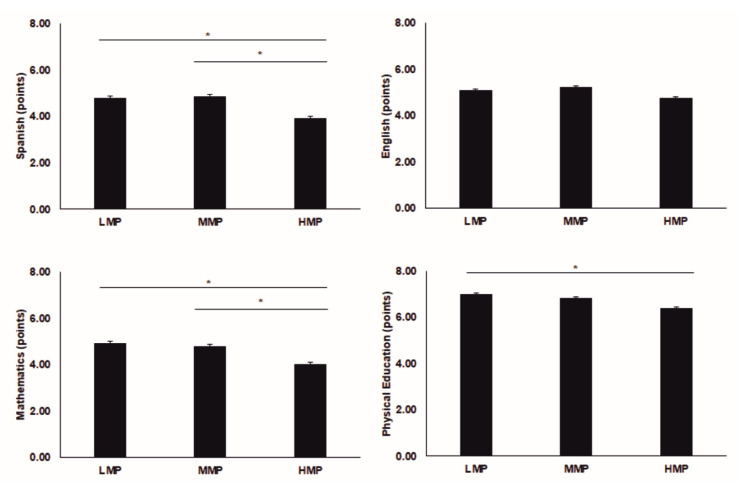
Results of academic performance. LMP = low use of the mobile phone; MMP = medium use of the mobile phone; HMP = high use of the mobile phone. * significant differences between groups; significant criteria *p* < 0.05.

Significant interactions between mobile-phone-use groups and gender were found for Spanish, English and Physical Education subjects. Spanish results for girls were 32.3% greater than for the boys’ group in the LMP group (*p* < 0.001). Girls showed 17.7% higher values in Spanish than boys in the MMP group (*p* = 0.001) and 46.22% higher in the HMP group (*p* = 0.006). English marks for girls were 10.7% higher than for boys in the MMP group (*p* = 0.019) and 25.5% greater in the HMP group (*p* < 0.001). No differences were described between boys and girls in mathematics and physical education marks. The group means are presented in Table 2. Without the assumption of the gender effect, significant differences were described for Spanish (*p* = 0.006), mathematics (*p* = 0.036) and physical education (*p* = 0.036), with higher values in the LMP and MMP than in the HMP group (Figure 3).

For boys, differences among mobile-phone-use groups were observed in academic performance. The Spanish marks in the MMP group were 30.6% greater than in the HMP group (*p* = 0.004). The physical education marks in the LMP group were 16.5% greater than in the HMP group (*p* = 0.009). No intergroup differences were found in mathematics and English. For the girls, no differences among mobile-phone-use groups were described (*p* > 0.05).

### 3.4. PF and Academic Variables Relationship

A positive correlation was found between physical fitness and academic performance (r = 0.359; *p* < 0.001). Physical education marks also showed a positive correlation with academic performance (r = 0.607; *p* < 0.001).

## 4. Discussion

This study describes the association between use of the mobile phone and PF and academic performance in high school students and the differences between both genders. The main findings of our study were: (i) The students with the highest use of mobile phon, without the effect of gender, showed worse results in PF and academic performance than in the LMP and MMP groups; (ii) Boy students presented higher values in PF than girl students when the use of the mobile phone was low or medium (LMP and MMP groups); (iii) English and Spanish academic results were better for girls than boys independently of mobile phone use; (iv) A positive correlation was described between physical fitness and academic performance. All of these results suggest that higher use of the mobile phone was related to worse results in physical fitness and academic performance in high school students—although longitudinal studies are warranted to confirm it—and also that the association with other variables such us socioeconomic status and physical activity should be investigated.

The results of our research show higher marks for boy students in most of the physical tests evaluated compared to girl students. These differences could be attributed to the action of testosterone, which has an important role in physical performance, especially in relation to strength [32]. Based on the premise that the maturation process is responsible for the gender differences in physical fitness, boy students should show better marks than girls in all the mobile-phone-use groups examined. Previous studies have reported greater than 55% gender-related differences in a similar abdominals test and upper-body-strength test [33]. However, contrary to this hypothesis, no differences were observed in the physical fitness test between boy and girl students in the HMP group. Therefore, maturity is not enough to provoke the gender-related differences in physical fitness and even less when, according with the EUROFIT guide [27], the marks are rated in a specific way for gender; therefore, other factors must be associated with the differences, such as daily time devoted to physical activity [20]. Previous research showed the relevance of physical-activity time per day in young athletes and its relationship with coordinative aspects [34]. Therefore, the greater use of the mobile phone for HMP students could provoke a decrease in physical-activity time and lead to a reduction of the gender-related differences. Finally, mental fatigue associated with mobile-phone use could also contribute to a decrease in physical fitness [35].

In our research we found intergroup differences in physical fitness for boy students but not for girls; thus, use of the mobile phone could affect them in a different way depending on gender. Boys in the HMP group showed lower values in physical fitness tests than those in the LMP and MMP groups. Currently, more than 85% of students have a mobile phone and described smartphones as an integral part of their daily lives [2]. This phenomenon could lead adolescents to have less free time for necessary physical activity. On the other hand, Stephens and Allen [36] found that appropriate use of the mobile phone supported by education was an effective tool to reduce sedentarism and obesity; the problem arises when previous studies support that the bulk of time students spend on their mobile phone is for leisure (~70%) and when they are using their mobile phone they are typically sitting (~87%) [37]. Previous research suggested gender-related different responses to the decrease in physical-activity time [20]. Lower values of physical-activity time regardless of exercise intensity reduced the boys’ physical fitness, while for girls the differences in physical fitness were in relation to the time spent in physical activity of high intensity [20]. Higher values of CERM (>20 points) showed a relationship with a greater level of addiction to the mobile phone [25]. In our study, although we did not find differences in CERM points between boy and girl students, the percentage of boys in the MMP and HMP groups was higher than that of girls (~69% vs. ~64%), contrary to previous research [23,25]. Therefore, the high percentage of boy students in the groups with a greater addiction to mobile-phone use could result in less physical-activity time and also could be the reason for the physical-fitness differences described among the boys’ groups.

Academic performance in students was evaluated based on criteria established by the curriculum for secondary education. From our results and according to previous research [4], there was an association between mobile-phone use level and academic performance, and those groups with lower levels of mobile-phone use, LMP and MMP, achieved higher academic results. In our study, girls showed better results than boys in language subjects (Spanish and English), and these differences did not change when the student level of mobile-phone use was factored in. The higher use of mobile phones for the boys could be the reason for the lower marks in academic performance. In addition, contrary to physical fitness, the gender-related differences did not disappear for the HMP group following the line described in a previous meta-analysis that showed better school-grade marks for girl students [21]. Finally, a significant correlation was found between physical fitness and academic performance, therefore preserving high levels of physical fitness could help to reduce the negative impact of mobile-phone use. Coinciding with our results, previous studies confirmed that those students with lower physical fitness and higher body fat obtained poor academic achievements [38,39].

This study has several limitations. First, the use of subjective methods, such as questionnaires, presents important limitations, because it is well known that self-perceptions in questionnaires tend to overestimate usage. Future research should provide specific and accurate analysis of mobile-phone use incorporating objective methodologies. Second, due to the differences in academic curriculums and assessments in secondary schools all around the world, it is difficult to establish comparisons in academic performance with other investigations. In addition, only common subjects were studied; therefore, new investigations are warranted to analyse the association between the use of mobile phones with performance in other subjects such as economics or history. Third, the individual physical-activity time was not recorded, although previous studies showed high correlations between physical fitness and physical-activity daily time. It is reasonable to think that a more in-depth analysis of physical activity, sports practice and socioeconomic status would have to be implemented, as well as the kind of apps used by the students, to gain insight into their roles as contributors to the problematic use of the mobile phone. Finally, the study design (cross-sectional study) did not allow establishment of causal effects; therefore, the results should be corroborated by longitudinal research to confirm the association between mobile-phone use and PF and academic performance. 

## 5. Conclusions

In conclusion, the results of the present study indicate that the groups of secondary school students with lower values of mobile-phone use outperform students with greater values both physically and academically. Boys showed better results for physical fitness than girls when mobile-phone use was low. The gender-related differences in physical fitness disappeared when mobile-phone use was high. Academic performance was higher for the girls’ group and did not appear to be conditioned by mobile-phone use. Finally, a positive correlation was described between physical fitness and academic performance.

## Figures and Tables

**Table 1:** Results of physical variables.

Group	Boys	Girls	Pairwise Comparisons (Gender)			Interaction (Gender × Mobile Use)	
			∆	95%-CI	ES	F	*p*
**Abdominals Test**							
LMP	7.06 ± 2.43	6.57 ± 2.36	0.49	−0.34 to 1.33	0.21	4.6	<0.001
MMP	6.86 ± 2.32 ^†^	6.16 ± 2.18	0.70	0.14 to 1.25	0.31		
HMP	5.32 ± 2.84 ^a,b^	5.47 ± 2.18	0.15	−1.35 to 1.05	0.06		
**Medicine Ball Throw**							
LMP	6.34 ± 2.24 ^†^	5.28 ± 1.86	1.06	0.29 to 1.82	0.51	11.3	<0.001
MMP	6.49 ± 2.52 ^†^	5.02 ± 1.68	1.37	0.97 to 1.98	0.69		
HMP	5.12 ± 2.55 ^a,b^	4.38 ± 1.35	0.74	−0.36 to 1.84	0.36		
**Broad Jump**							
LMP	6.97 ± 2.92 ^†^	5.46 ± 2.96	1.51	0.56 to 2.46	0.51	10.0	<0.001
MMP	6.76 ± 2.85 ^†^	5.02 ± 2.19	1.74	1.10 to 2.37	0.68		
HMP	5.40 ± 2.57 ^a^	4.60 ± 2.02	0.80	−0.57 to 2.16	0.35		
**50 m Sprint**							
LMP	6.84 ± 2.52 ^†^	5.74 ± 2.58	1.10	0.23 to 1.97	0.43	5.1	<0.001
MMP	7.02 ± 2.42 ^†^	5.90 ± 2.37	1.12	0.54 to 1.70	0.47		
HMP	6.00 ± 2.00	5.62 ± 2.35	0.38	−0.87 to 1.63	0.18		
**Deep Trunk Flexion**							
LMP	7.23 ± 2.58	7.46 ± 2.47	0.23	−1.08 to 0.62	0.09	3.3	0.007
MMP	7.19 ± 2.38	6.72 ± 2.09	0.47	−0.90 to 1.03	0.21		
HMP	5.56 ± 2.72 ^a,b^	6.50 ± 2.08	0.94	−2.15 to 0.27	0.39		
**Agility Test**							
LMP	8.23 ± 2.08 ^†^	7.30 ± 2.57	0.97	0.12 to 1.74	0.40	5.5	<0.001
MMP	8.19 ± 2.18 ^†^	7.01 ± 2.36	0.81	0.64 to 1.72	0.52		
HMP	7.16 ± 1.65	7.09 ± 2.06	0.07	−1.09 to 1.23	0.04		

Data are expressed as means ± SD; LMP = low use of the mobile phone; MMP = medium use of the mobile phone; HMP = high use of the mobile phone; 95% CI = 95% confidence intervals for the difference; ES = effect size; ^a^ significant differences from LMP group; ^b^ significant differences from MMP group; ^†^ significant differences between boys and girls; significant criteria *p* < 0.05.

**Table 2:** Results of academic variables.

Group	Boys	Girls	Pairwise Comparisons (Gender)			Interaction (Gender × Mobile Use)	
			∆	95%-CI	ES	F	*p*
	**Spanish**						
LMP	4.16 ± 2.20 ^†^	5.50 ± 2.13	1.34	−2.05 to −0.64	0.62	8.7	<0.001
MMP	4.48 ± 2.12 ^†^	5.27 ± 1.94	0.79	−1.27 to −0.32	0.39		
HMP	3.11 ± 1.57 ^b,†^	4.54 ± 2.02	1.43	−2.46 to −0.42	0.79		
	**Mathematics**						
LMP	4.69 ± 2.23	5.19 ± 2.22	0.50	−1.24 to 0.23	0.23	2.0	<0.001
MMP	4.71 ± 2.19	4.82 ± 2.01	0.11	−0.60 to 0.39	0.05		
HMP	3.93 ± 2.04	4.06 ± 1.68	0.07	−1.21 to 0.94	0.07		
	**English**						
LMP	4.86 ± 2.09	5.32 ± 1.99	0.46	−1.12 to 0.21	0.23	3.1	0.010
MMP	4.96 ± 1.99 ^†^	5.49 ± 1.78	0.53	−0.97 to −0.87	0.28		
HMP	4.14 ± 1.72 ^†^	5.20 ± 1.83	1.06	−0.20 to −0.99	0.60		
	**Physical Education**						
LMP	7.03 ± 1.59	6.90 ± 1.57	0.13	−0.38 to 0.64	0.08	2.3	0.047
MMP	6.69 ± 1.38	6.92 ± 1.52	0.23	−0.57 to 0.12	0.16		
HMP	6.04 ± 1.29 ^a^	6.66 ± 1.59	0.62	−1.36 to 0.12	0.43		

Data are expressed as means ± SD; LMP = low use of the mobile phone; MMP = medium use of the mobile phone; HMP = high use of the mobile phone; 95%-CI = 95% confidence intervals for the difference; ES = effect size; ^a^ significant differences from LMP group; ^b^ significant differences from MMP group; ^†^ significant differences between boys and girls; significant criteria *p* < 0.05.

## Data Availability

The data presented in this study are available on request from the corresponding author. The data are not publicly available due to privacy restrictions.
